# EPOS: EEG Processing Open-Source Scripts

**DOI:** 10.3389/fnins.2021.660449

**Published:** 2021-06-07

**Authors:** Johannes Rodrigues, Martin Weiß, Johannes Hewig, John J. B. Allen

**Affiliations:** ^1^Department of Psychology I: Differential Psychology, Personality Psychology and Psychological Diagnostics, Julius-Maximilians-Universität Würzburg, Würzburg, Germany; ^2^Department of Psychology, University of Arizona, Tucson, AZ, United States

**Keywords:** EEG, electroencephalography, event-related potentials-ERP, EEG processing, EEG preprocessing, EEG frequency band analysis

## Abstract

**Background:**

Since the replication crisis, standardization has become even more important in psychological science and neuroscience. As a result, many methods are being reconsidered, and researchers’ degrees of freedom in these methods are being discussed as a potential source of inconsistencies across studies.

**New Method:**

With the aim of addressing these subjectivity issues, we have been working on a tutorial-like EEG (pre-)processing pipeline to achieve an automated method based on the semi-automated analysis proposed by Delorme and Makeig.

**Results:**

Two scripts are presented and explained step-by-step to perform basic, informed ERP and frequency-domain analyses, including data export to statistical programs and visual representations of the data. The open-source software EEGlab in MATLAB is used as the data handling platform, but scripts based on code provided by Mike Cohen (2014) are also included.

**Comparison with existing methods:**

This accompanying tutorial-like article explains and shows how the processing of our automated pipeline affects the data and addresses, especially beginners in EEG-analysis, as other (pre)-processing chains are mostly targeting rather informed users in specialized areas or only parts of a complete procedure. In this context, we compared our pipeline with a selection of existing approaches.

**Conclusion:**

The need for standardization and replication is evident, yet it is equally important to control the plausibility of the suggested solution by data exploration. Here, we provide the community with a tool to enhance the understanding and capability of EEG-analysis. We aim to contribute to comprehensive and reliable analyses for neuro-scientific research.

## Introduction

The electroencephalogram (EEG) is one of the most important tools in both applied and clinical neurophysiology as it offers a high temporal resolution and a high safety due to its non-invasive application (Cohen, 2017). The central properties of this measurement instrument for electrical activity, first described by Berger (1929), are frequency (oscillations per time period) and amplitude (maximum value of an oscillation during one period). Years later and despite many technical developments of the systems and the existing software for subsequent processing, there are still major problems in the replicability of findings in EEG research due to methodological variations across laboratories (Bishop, 2007). However, the problem is not limited to EEG research. Recently an article was published which shows how much flexibility in preprocessing affects the results of research using Magnetic Resonance Imaging (Botvinik-Nezer et al., 2020), which has major implications for scientific conclusions.

The EEG signal is strongly affected by sources of interference, which are caused by the application of the electrodes (e.g., electrode displacement), the experiment itself (e.g., flickering frequencies) or by the activity of the participants, partly in interaction with the previous factors (e.g., eye-movements or muscle activity). These unwanted signals can be much larger than the actual signal of interest and therefore massively interfere with the measurement of electrophysiological correlates of neural activation if the artifacts are not corrected (e.g., Cuevas et al., 2014). The resulting corrections and the further processing of this data raises obstacles to replicability. The replication crisis in psychophysiology was addressed by Larson and Moser (2017) in a special issue entitled “Rigor and Replication: Toward Improved Best Practices in Psychophysiological Research” in the International Journal of Psychophysiology. Included are, among other things, contributions on general improvement of rigor (Baldwin, 2017) reliability analysis of ERPs (Clayson and Miller, 2017) replication of time-frequency data or sample size calculation for electrophysiology (Larson and Carbine, 2017).

In current EEG research, there are high degrees of freedom for the researchers in terms of analysis but also in terms of reporting in publications, which leads to an increase in the false positive rate of research findings (Simmons et al., 2011). Several sets of guidelines for data consistency and replicability have been published (Pivik et al., 1993; Picton et al., 2000; Keil et al., 2014), but tools for ensuring consistent processing are still needed (for the most recent approach, see Debnath et al., 2020). For EEG data, this means a flexible choice of time-window, frequency band, filtering specifications, electrodes, reference, measurement, artifact rejection, and outlier exclusion. Most researchers use different filters, references, and criteria for artifact removal prior to the actual analysis for a variety of (good) reasons. Nevertheless, this process is by no means standardized, making it almost impossible to combine data sets from different data sources for analysis without preprocessing them jointly. Consequently, Keil et al. (2014) pointed out that standardization and automation in the processing of electrophysiological data will be indispensable.

To preprocess EEG data, various pipelines have been developed in the recent past to address the growing need for standardization. The PREP pipeline (Bigdely-Shamlo et al., 2015) provides a standardized method to remove line-noise (Mullen, 2012) and an average referencing to detect and interpolate noisy channels. However, PREP focuses only on experiment-related artifacts and not on individual artifacts like eye-blinks. The Harvard automated preprocessing pipeline (HAPPE; Gabard-Durnam et al., 2018) adds an independent component analysis (ICA) and uses a Multiple Artifact Rejection Algorithm (Winkler et al., 2011) to correct artifacts. But, according to the authors, this pipeline is not suitable for the analysis of event-related potentials. The Computational Testing for Automated Preprocessing (CTAP; Cowley et al., 2017) toolbox has a similar approach to HAPPE, but allows the user to compare the outcomes of different preprocessing pipelines. Moreover, the Batch Electroencephalography Automated Processing Platform (Levin et al., 2018) was created, which aims to simplify and standardize the replication of existing studies through a collection of preprocessing pipelines applied to new data sets. In addition, Automagic (Pedroni et al., 2019) was introduced, a wrapper toolbox that combines common preprocessing methods. Automagic uses the PREP pipeline per default and adds further processing steps afterward. The automatic pre-processing pipeline (Ramos da Cruz et al., 2018) for large datasets proved to be an efficient and reliable method for both resting state and evoked EEG, which was tested for both clinical and healthy participants. Finally, the Maryland analysis of developmental EEG (MADE) pipeline was recently published (Debnath et al., 2020). This pipeline focuses on the standardized and automatic preprocessing of data from pediatric populations using EEGLAB.

The reason why we came up with our own approach was that most of the previous mentioned pipelines focus on some specific concepts and parts of the pre-processing, while our approach tries to orient on and extend the principles provided by Delorme and Makeig as they advised to preprocess the data up to 2019^1^. However, where Makeig and Delorme suggested semi-automatic detections or visual detections of the data, we suggest standardized selection criteria based on statistical outlier detection or algorithm and machine-learning based artifact selection (Winkler et al., 2011) and therefore come to replicable and standardized (pre-)processing results. Our work is particularly aimed at researchers in the introductory phase by presenting one possible approach that the authors can recommend based on the current consensus of the community. The aim of this paper is therefore to present standardized and automated EEG processing open-source scripts (EPOS). In particular, we want to offer newcomers to EEG research a step-by-step tutorial that may help to produce openly communicated analyses that can be replicated by other researchers, since all relevant information is given and can be reproduced. In this context, we aim to facilitate and improve the decisions where the user has to define criteria (e.g., electrode sites for ERPs or frequencies) by integrating a set of visualizations. We do not want to focus on individual processing steps, but rather provide a comprehensible tutorial script of the entire process after data collection up to the extraction of the final data for analysis. Except for the screening of complete data sets, no intervention based on subjective non-documented or non-replicable decisions of individuals in the artifact cleaning will be performed, as only replicable and standardized criteria may be chosen. There are very early findings suggesting that algorithmic approaches exceed individual valuation standards, so that actuarial approaches, once validated, should be preferred over subjective judgments (e.g., Dawes et al., 1989). In a meta-analysis, Grove et al. (2000) were even able to show that the mechanical prediction or more accurately statistically defined prediction criteria performed significantly better than the clinical prediction in 33–47% of the studies examined, while the clinical prediction was more accurate in only 6–16% of the studies examined. Additionally, subjective standards vary inter-individually as well as intra-individually, while an algorithm has a replicable performance. Hence, we provide a (pre)-processing pipeline that is based on mechanical and reproducible criteria to avoid subjective variability.

## Materials and Equipment

We provide scripts for data export for statistical analysis in other software as well as for the visualization (ERPs, time-frequency plots, topographical maps) of electrophysiological data, to control for plausibility of the standardized solutions in EEG-analyzes^2^.

The preprocessing pipeline proposed here will need the following software toolboxes: EEGLAB (Delorme and Makeig, 2004) with the plugins IClabel (Pion-Tonachini et al., 2019), ADJUST (Mognon et al., 2011), MARA (Winkler et al., 2011), SASICA (Chaumon et al., 2015), and the CSD Toolbox (Kayser, 2009; Jürgen Kayser and Tenke, 2006a,b) or the CSD transformation provided by Cohen (2014). All these packages run on MATLAB (Matlab, 2011), but some attempts are done to convert these packages to Octave (Eaton, 2002), an open source version of MATLAB.

## Methods

In the following, we will first present the proposed standard preprocessing pipeline for EEGLab (Delorme and Makeig, 2004) that was provided up to 2019 on https://sccn.ucsd.edu/wiki/Chapter_01:_Rejecting_Artifacts and then present and justify our changes and extensions. This pipeline is not to be seen as the new method for all applications that can simply be thrown at any type of data, but it is a proposal for a reproduceable analysis, that may help beginners in several cases of data-(pre)-processing. Some suggestions seem to be debatable at first glance (e.g., filtering after an initial “segmentation” and not before, which may cause edge artifacts, or filtering with 1 Hz if interested in low frequency bands). However, when reading through the suggestions in detail and also consulting the explanation in the respective scripts, users might note that some assumed problems are not given when following the suggestions in principle (e.g., taking long first data segments to avoid filtering the entire dataset or extracting unfiltered data ICs if users are interest in low frequency bands).

### Preprocessing According to EEGLab

A summary of the previously proposed preprocessing steps is:

Rejection based on independent data components:

Step 1. Visually reject unsuitable (e.g., paroxysmal) portions of the continuous data.
Step 2. Separate the data into suitable short data epochs.
Step 3. Perform ICA on these epochs to derive their independent components.
Step 4. Perform semi-automated and visual-inspection based rejection of data epochs on the derived components.
Step 5. Visually inspect and select data epochs for rejection.
Step 6. Reject the selected data epochs.
Step 7. Perform ICA a second time on the pruned collection of short data epochs.
Step 8. Inspect and reject the components. Note that components should NOT be rejected before the second ICA, but after.

### Preprocessing According to EPOS

As mentioned above, we tried to replace subjective un-replicable influences with standardized approaches.

First, we would like to point out that a good and standardized preprocessing can be worth a lot, but clean EEG data recording is essential (“garbage in → garbage out”). Therefore, the first step that is never mentioned but which is essential is to take your time with the data acquisition and apply the EEG caps/electrodes responsibly and with care.

**FIGURE 1:**
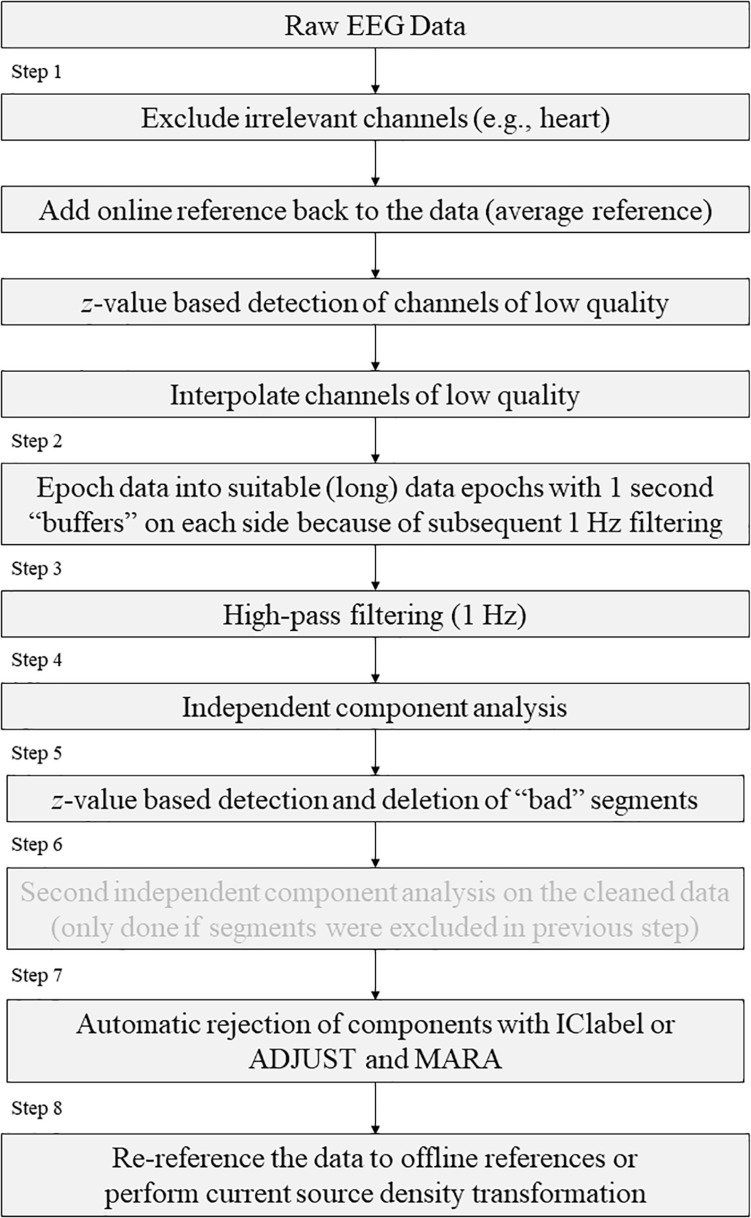
Schematic representation of the preprocessing steps as recommended by the EPOS pipeline.

The new (pre-)processing “chain” that is proposed based on the previous mentioned chain, as illustrated in Table 1 and Figure 1:

**TABLE 1:** Comparison of the individual steps in the preprocessing pipelines according to EEGLab and EPOS.

**EEGLab**	**EPOS**
Visually reject unsuitable portions of the continuous data.	Statistically detect and interpolate channels of low quality.
Separate the data into suitable short data epochs.	Separate the data into suitable data epochs.
Perform ICA on these epochs	High-pass filtering
Semi-automated and visual inspection-based rejection of data epochs on the derived components.	Perform first ICA
Visually inspect and select data epochs for rejection.	Detection and deletion of bad segments based on z-value detection on ICs
Reject the selected data epochs.	Second ICA
Second ICA	Automatic inspection and rejection of the components using ICLabel or ADJUST and MARA with SASICA
Inspect and reject the components.	Re-reference (to CSD)

Step 1. Statistically detect and interpolate channels of low quality.
Step 2. Separate the data into suitable data epochs.
Step 3. High-pass filtering.
Step 4. First independent component analysis.
Step 5. Detection and deletion of bad segments based on *z*-value detection on ICs
Step 6. Second independent component analysis.
Step 7. Automatic inspection and rejection of the components using either ADJUST and MARA with SASICA or ICLabel.
Step 8. Re-reference (to current source density CSD).

### Processing According to EPOS

**FIGURE 2:**
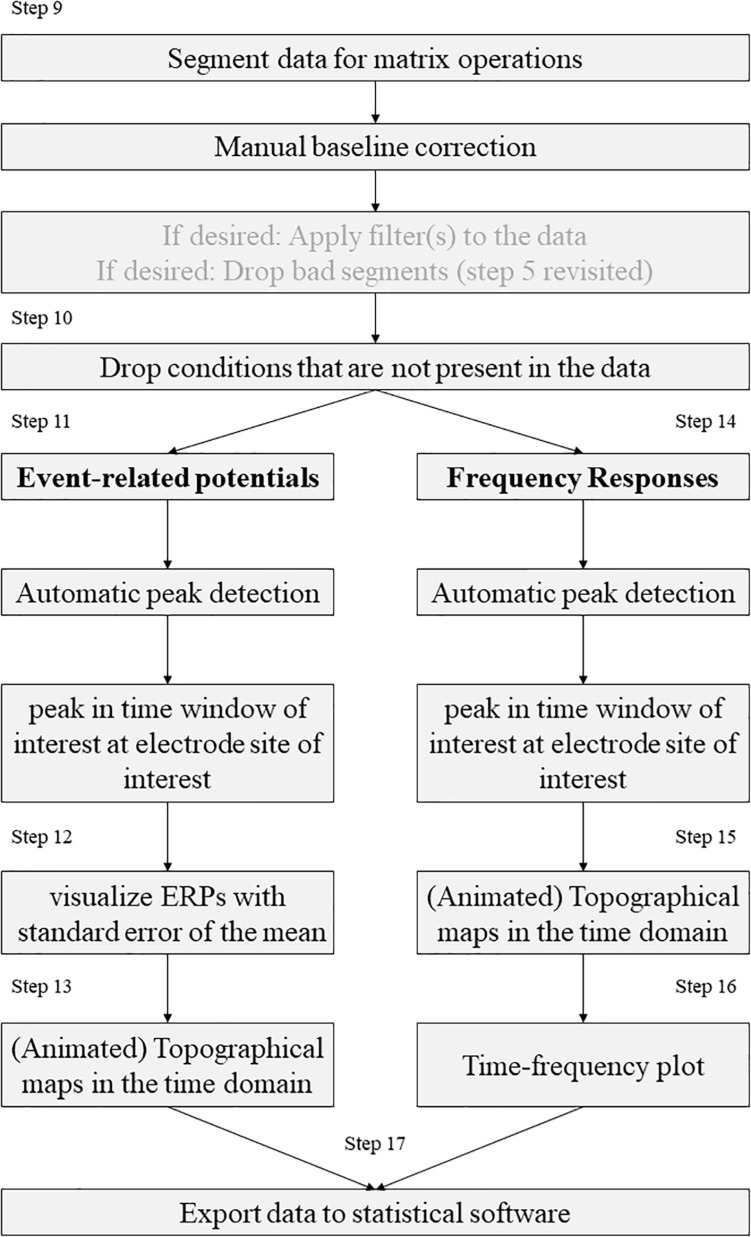
Schematic representation of the processing steps as recommended by the EPOS pipeline.

After the pre-processing of the EEG data has been completed at this point in a standardized and automated procedure, we will describe the further processing of the data in the following steps (see Figure 2). For this purpose, nine steps are performed, some of which are optional, depending on the experiment, data set and personal preferences. The MATLAB addons *boundedline* (Kearney, 2020) and *export_fig* (Altman, 2020) are required for creating graphics and exporting data. Also, the wavelet function based on the code provided by Cohen (2014) and edited by John J.B. Allen and Johannes Rodrigues is required to analyze time frequency results. To perform single trial analyses for frequencies later, an adjustment was made to the frequency extraction functions of Cohen (2014). Otherwise these functions were implemented as described by Cohen (2014).

Step 9. Segment the data for analysis.
Step 10. Drop the cases that are not present (for example in free choice paradigms).
Step 11. Automatic peak detection in a given time-window in EEG signal.
Step 12. Compute and visualize event-related potentials.
Step 13. Topographical maps (Topoplots) in the time-domain.
Step 14. Automatic peak detection in a given time window in frequency responses.
Step 15. Topographical maps for frequency responses.
Step 16. Time-frequency plot for a specific electrode in a broad frequency window.
Step 17. Export the data to statistical software.

## Results

### Preprocessing According to EPOS

#### Step 1: Statistically Detect and Interpolate Channels of Low Quality

**FIGURE 3:**
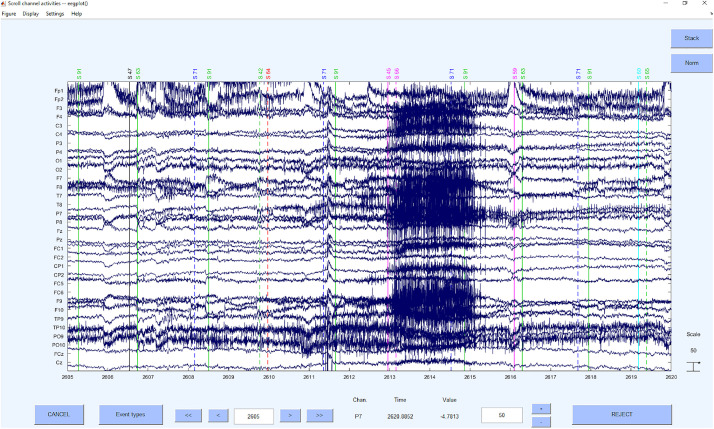
Raw data example (not too good data).

This step is based on the raw data (see example in Figure 3) and detects and excludes channels with a very low signal to noise ratio. These channels will be interpolated and therefore will not contribute with their signal to the signal that will be processed further.

**FIGURE 4:**
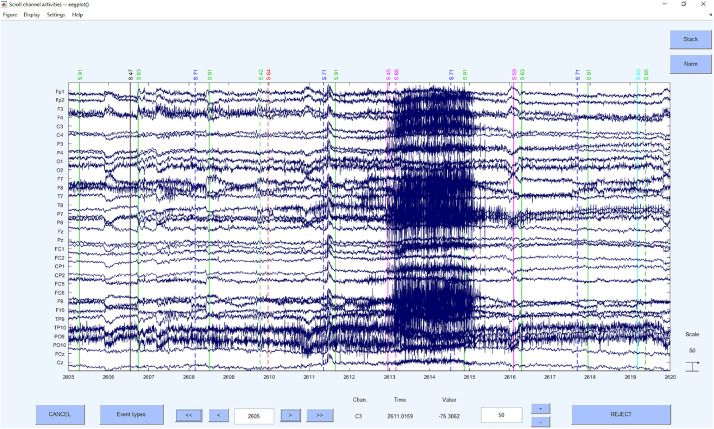
Interpolated data channels.

Before a detection of the “bad” channels can be done, only the relevant channels have to be selected, Channels that are to be ignored for the following processing steps are for example, heart electrodes or skin conductance measurements, that may be (in-)directly related to brain waves but not of the same structure as the EEG signal. After selecting the EEG electrodes, the online reference should be added back to the data so that this electrode can also be used for further analyses or interpolated in the case of too much noise in this channel. As a reference system is required while also retaining the online reference as a channel, we use average reference to further process the data, although it is not a preferable “final” reference scheme and can still be changed in later steps. The average reverence is very useful at this processing stage, if one has a sufficient amount of electrodes that cover the scalp fields sufficiently (Junghöfer et al., 1999). If this is not given, one might introduce a bias based on the electrode distribution to the data. If other electrodes are being used as offline reference, one loses the electrode for interpretation in the data. Therefore, we would not recommend this approach, but if not possible otherwise, also other reference electrodes (for example linked mastoids) can be used right away here, losing the respective electrodes in the following processing steps. As mentioned above, we may later change the reference, even to a CSD reference, but we may not use this reference for automatic IC detections based on MARA and ADJUST or IClabel, as they are not trained with these spatially filtered parameters and therefore come to very wrong conclusions. After the first re-referencing to regain the online reference channel, as the first preprocessing step the “bad” channels are detected and interpolated using statistical criteria. We use a detection based on *z*-values. The probability, kurtosis and spectrum are detected according to the outlier criterion *z* > 3.29 (Tabachnick and Fidell, 2007) for univariate statistical outliers. For the spectrum we use as frequency range 1–125 as suggested by Makeig and Delorme (see text footnote 1). The interpolation of the bad channels is done instead of a mere exclusion because of the irregularities of the matrices that would be introduced into the data structure and that would interact with later pre-processing and processing steps. Of course, the information of the interpolated channel is lost and therefore the rank of the matrix is reduced from information perspective, but for practical reasons, the structure of the data can be retained (see example in Figure 4).

To get into the function details of this step, the EPOS uses the EEGLAB *pop_select* function to select the channels, which are to be ignored for the preprocessing. The function *pop_chanedit* makes space for the online reference, which will be added back to the data. Using *pop_reref* the data is re-referenced to the average of all electrodes. The included channels for re-referencing depend on the montage and must be adjusted in our script. Finally, the EEGLAB function *pop_rejchan* performs the detection of distorted channels according to the statistical criteria and *pop_interp* interpolates the resulting channels of poor data quality.

#### Step 2: Separate the Data Into Suitable Data Epochs

**FIGURE 5:**
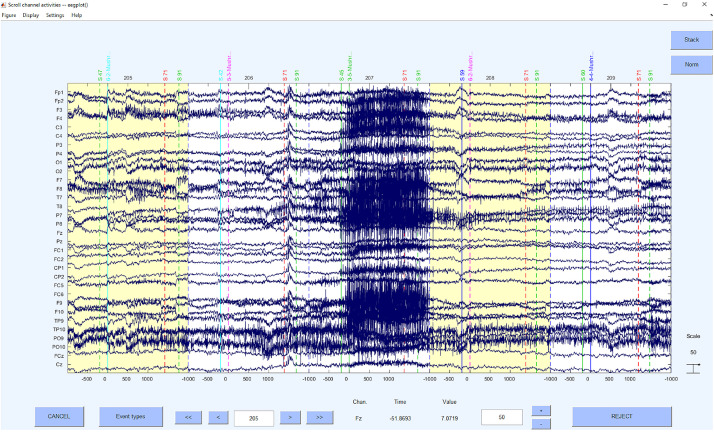
First segmentation. The marked segments are visual aids to evaluate later steps.

The next step is to slice the data into suitable “first” data epochs which will later be segmented into the “real” segments but should be rather long as their purpose is to be the database for the ICA. However, the length of a segment should also be chosen depending on the homogeneity of a trial. It is important to choose the data epochs as long as possible, since the following ICA provides a better solution for longer data periods. At the same time, the segments should be as short as possible, since the ICA solution leads to noisier and more unspecific ICs for different tasks and therefore to a less sensitive *z*-value based artifact detection concerning the exclusion of the segment. In summary, the signal needs to be long enough to obtain a reliable measure and short enough to account for the rather non-stationary nature of EEG signals (Korats et al., 2012). Therefore, we recommend either to segment the whole trial (if the trial is long enough and not too many different phases are present, or the trials are short and homogeneous) or to segment parts of an experiment. The segments in this phase may have a length of 8–20 s (e.g., Möcks and Gasser, 1984), depending on the data quality and the task. If you expect rather noisy data with huge artifacts that are rather short, also a short segment length of down to 2 seconds can be advisable. However, in the later step of processing the data (step 9) the rejection of smaller segments can be implemented. Hence, we recommend longer segments at this stage. Please keep in mind that a time window for the baseline correction is also necessary and should be included (x seconds before the marker/event of interest). For frequency analysis, more space is needed on both sides of a segment, since “edge effects” (i.e., distortions or transient effects resulting from a time-window larger than the time window for which data are available) can occur (Debener et al., 2005; Herrmann et al., 2005; Roach and Mathalon, 2008). The same applies to filters, which can also lead to edge effects. To avoid these artifacts later, we would recommend 1 s of “buffer time” on the segments on each side, as we will apply a 1 Hz filter in the next step and the 1 Hz filter may produce “filter rippling” up to 1 s. In homogeneous data, overlapping segments can easily be used, although one has to take into account that this might also alter the data. An example for the segmented data can be seen in Figure 5.

Getting more into function detail, we use the EEGLAB function *pop_epoch* to slice the data into segments of a suitable length, depending on the experiment and its trial duration.

#### Step 3: High-Pass Filtering

As a third step we apply a 1 Hz high pass filter to the data. This is done to get a more stable ICA solution as no low frequency shift is present (Winkler et al., 2015). Furthermore, after extensive testing of the influence of different filters on the performance of MARA (Winkler et al., 2011), we found that MARA works best with only the 1 Hz filter. A 2 Hz filter, for example, will not correct side eye movements as good as if it was filtered with 1 Hz and data filtered to the power spectrum between 2 and 39 Hz (as recommended in the MARA manual) will not correct muscular activation as well as it is done without the 39 Hz filter. At this point we would also like to remind that every filter changes the data, although the filtering at this point is only used to get a better basis for the ICA and artifact rejection based on the automatic artifact IC detection of MARA (Winkler et al., 2011) and ADJUST (Mognon et al., 2011). As previously stated, there might occur edge artifacts (filter rippling). These artifacts are very prone to occur in short data epochs, as they are on the edges of the filtered data. Because of this problem, one normally recommends filtering unsegmented continuous data, instead of “segmented” data. However, as the “segmentation” we performed in step 2 is basically a selection of a large continuous data part with sufficient edges for the occurring filtering artifacts, instead of only the shortest data part of interest, one may start the filtering at this step, getting a quicker and more efficient filtering process, because only parts of the data need to be filtered and not the entire dataset. Also as mentioned above we recommended 1 s data “buffers” on the large data “segments” to avoid filter rippling in the relevant parts of the data with a 1 Hz high-pass filter. As a general comment on filters, one has to keep in mind, that filters only attenuate the frequency bands they are designed to work on and are not built for a complete dampening of the respective frequency responses. This may result in residual artifacts of very large frequency artifacts, if the dampening curve is applied with an inappropriate filter order (i.e., a very large muscular artifact will have effects even after the filtering). To close this general comment about filters follows a quick reiteration of the different filter types that may be applied: Notch [deletes the target frequency, e.g., 50 Hz (AC in Europe)], low-pass/high-cut (all frequencies below the target frequency will be attenuated), high-pass/low-cut (all frequencies above the target frequency will be attenuated) and bandpass (combines low-pass plus high-pass). It is important to note, that some research interests are in the frequency band below 1 Hz and therefore usually apply much lower high-pass filters (for example 0.001 Hz if interested in slow waves). However, as there is the opportunity to write the IC solutions back to the unfiltered data, users may apply the 1 Hz filtering at this point to get a good performance of ICA and the MARA algorithm or similar algorithms like ICLabel. Of course, one may also use different automatic and standardized algorithms to detect artifact components, that perform better with other filter solutions or apply for specific data that may have unique features (e.g., Rodrigues et al., 2020c).

**FIGURE 6:**
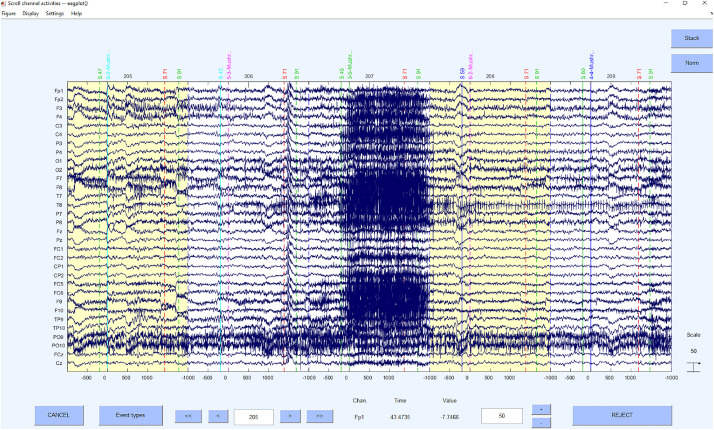
Filtered data.

Again, providing the function details of this step, we apply the EEGLAB function *pop_eegfiltnew*, for which the toolbox firfilt (Andreas Widmann) is required. Depending on the data segments, the filter order for the 1 Hz low-pass filter must be adjusted in the script. An example for filtered data can be seen in Figure 6.

#### Step 4: First Independent Component Analysis

In the next step we apply an independent component analysis to the data. The ICA is a statistical linear decomposition of the signal into independent components, each of which contributes as much specific information as possible to the data (Makeig et al., 2004). Thus, each electrode provides data that is assigned to a source/sensor. The ICA decomposes the linearly mixed sources at the sensor level into independent components (Bell and Sejnowski, 1995) and we get as many components as there are sources (i.e., 64 independent components with 64 electrodes). As a result, ICA separates the actual electronic brain signal from non-brain artifacts such as eye movements or muscle activity. The ICA is a so-called “blind” separation technique and therefore does not guarantee meaningful results (Jung et al., 2000). Not every extracted component is equally plausible and depends strongly on the data quality and the specific ICA algorithm used. Depending on the interpolation, however, less information is obtained for each interpolated channel. In order to avoid “ghost-ICs” that do not carry meaningful information, we can limit our ICs to a specific number that equals the “rank of the matrix,” in our case the channels that are not extrapolated and therefore still carry information (see e.g., Miyakoshi, 2021). The resulting ICs also have a time course and a frequency distribution as channels have. But as the topographical order of the channels is dissolved a new topographical projection is provided for each IC. Based on these features, artifact detection can be performed, either only using parts of this information (time course, topography, frequency response) or all together. In step 5, we will only use parts of the information neglecting the topography of the ICs to select artifact segments, while in step 7, the automatized machine learning, and criteria-based algorithms are using all information in order to select artifact ICs.

To perform the ICA, we use the command *pop_runica* in EEGLAB (Delorme and Makeig, 2004; Makeig et al., 2004).

#### Step 5: Detection and Deletion of Bad Segments Based on *z*-Value Detection on ICs

Now the bad segments are selected and deleted based on a *z*-value detection on the ICs. As in the first step, the criterion of *z* > 3.29 (Tabachnick and Fidell, 2007) for the probability and kurtosis is applied on the channel basis. The reason for this step is to increase the data quality to be able to clean up artifacts even better with the following second ICA. On global level we used a very high *z*-value threshold of *z* = 20 to only correct for very huge artifacts and to prevent the overcorrection of different signal components. This approach was recommended by Delorme and Makeig (2004), that was provided up to 2019 on https://sccn.ucsd.edu/wiki/Chapter_01:_Rejecting_Artifacts because of the higher sensitivity to “extraordinary” not regularly appearing artifacts (e.g., singular hiccup) than a merely channel *z*-value based artifact detection. However, if the data is rather noisy in general and artifacts with long durations are expected, we would also recommend to use a global threshold of *z* = 3.29 to detect the segments that need to be rejected.

**FIGURE 7:**
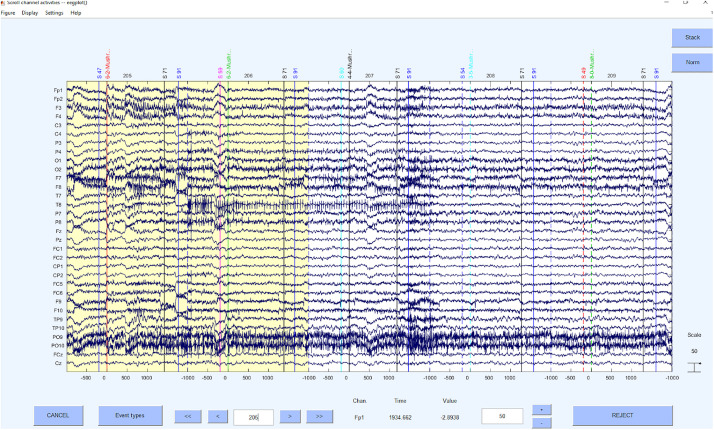
Bad segments excluded.

Concerning the underlying functions, the EEGLAB command *pop_jointprob* was used to reject the probability and *pop_rejkurt* for the kurtosis. The selected segments were removed using *pop_rejepoch*. An example for a rejection of segments can be seen in Figure 7.

#### Step 6: Second Independent Component Analysis

This second ICA is now performed on the data cleaned for poor segments. This step is only performed if at least one bad segment was detected and rejected. The goal is to achieve a better signal-to-noise-ratio by identifying artifact driven components containing no relevant signal. These artifact components will be deleted to compute signal that only consist of non-artifact data or that is at least less artifact polluted. Therefore, once again a signal decomposition is performed leading to the previously mentioned information in the ICs. After the ICA we select the ICs that represent signal and those that represent noise. Again, we use the EEGLAB function *pop_runica.*

#### Step 7: Automatic Inspection and Rejection of the Components Using ICLabel or ADJUST and MARA With SASICA

In the seventh step, the resulting components are automatically inspected and rejected. In a first more traditional approach, we were using ADJUST (Mognon et al., 2011) and MARA (Winkler et al., 2011) with SASICA (Chaumon et al., 2015). SASICA serves as EEGLAB plugin, which contains various artifact correction algorithms from different researchers (e.g., Fully Automated Statistical Thresholding for EEG artifact Rejection (FASTER), Nolan et al., 2010, ADJUST, and MARA).

ADJUST uses algorithms based on temporal and spatial filters to identify mainly (but not exclusively) artifacts caused by eye movements. These include blinks, horizontal and vertical eye movements, but also generic discontinuities. The algorithm uses an expectation-maximization-based approach to automatically detect the threshold of spatio-temporal properties of different artifact types and classify them accordingly (Wu et al., 2018). MARA combines different measures to automatically classify ICs as artifacts via a linear machine learning algorithm. In summary, two spatial, one temporal, and three spectral features provide the measures for the best classification results. These different classification components are described in detail in the original paper. MARA is not designed to detect a specific artifact type, but rather is variable to detect eye artifacts, muscle artifacts, the heartbeat, or loose electrodes. Regarding MARA, we have already set the reference to average and filtered the data with 1 Hz. MARA has shown to perform well in the automatic classification of artifacts (Winkler et al., 2011, 2014). Although trained and experienced EEG researchers may have an even better ability to distinguish signal from artifact components, automatic artifact correction algorithms have an increased reliability of artifact removal exceeding the human raters.

**FIGURE 8:**
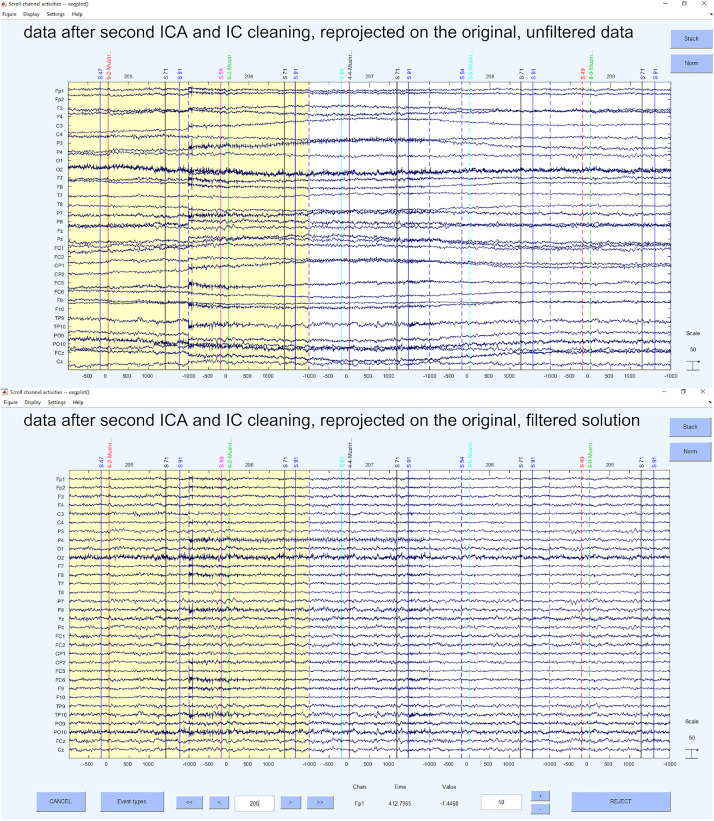
Data after second ICA and IC cleaning, reprojected on the original, unfiltered data and filtered data solution.

After we have configured the specific options (i.e., ADJUST or MARA marked it bad) in the script, we perform the automatic removal of the ICs with the EEGLAB command eeg_SASISCA. Using the marking of an artifact by ADJUST or MARA is a very conservative approach because it rejects as many ICs as possible. However, as ADJUST and MARA are selectively strong for certain artifacts (e.g., ADJUST is rather strong in detecting heart-beat related shifting artifacts that MARA tends to miss while MARA is more sensitive in detecting very noisy components) the selected method leads to less artifact prone data, yet being a very strict approach concerning mixed components. We also provide code that writes the ICA solution back to the unfiltered EEG data. Therefore, we project the ICA solution that is based on the automatic selection by MARA onto the original data without including the necessary preprocessing steps to achieve them. This leads to dropping the preprocessing artifacts that were introduced to gain an optimal performance of the standardized preprocessing. Hence, we gain a projected solution of the ICA, which might not be the identical solution that we would have gotten with an ICA on the raw data, but that is a projection of the optimally prepared data matrix for automatic IC selection on the raw data matrix. Please keep in mind, that we only recommend these packages because they are openly available and provide replicable results. The respective setting of these software packages might not fit for every need in detail and we highly encourage to still use other replicable solutions that may select ICs (Rodrigues et al., 2020c), as long as you provide these solutions and the important needed information to understand the procedure to other researchers and therefore guarantee the replicability of your analyses. Examples for the IC cleaned data can be seen in Figure 8, for the projected unfiltered data and the filtered data solution.

As an alternative to using MARA and ADJUST, we have implemented a more recent toolbox as a means of automated IC selection, called ICLabel. This classifier outperforms previous publicly available automatic IC component classification method for all measured IC categories, while calculating these labels ten times faster, as demonstrated by a comparison with other publicly available EEG IC classifiers (Pion-Tonachini et al., 2019). ICLabel is used to classify ICs into the seven categories of “brain,” “eye,” “muscle,” “heart,” “chan_noise,” “line_noise,” and “other.” To classify these components, ICLabel examines the spatio-temporal measure in the ICLabel dataset, which contains over 200,000 ICs from more than 6,000 EEG recordings. The scope of this labeled classification set used in the ICLabel learning implementation is accessed through the ICLabel website, which applies crowd-sourcing strategies to collect IC labeling data from experts. The ICLabel dataset used to train and evaluate the ICLabel classifier includes EEG datasets from a broad range of paradigms to achieve accuracy across all EEG recording conditions. After storing a ICA-decomposed dataset in a variable (e.g., “EEG”), ICLabel can be used by entering *EEG* = *iclabel(EEG)*, and IC classification results can be obtained by *EEG.etc.ic_classification.ICLabel.classifications*. Our “default” selection criterion linked to IClabel is rather inclusive, as we compare the probability of the signal with the artifact probabilities. If the signal is more likely to constitute the component, it is accepted as signal. Note, however, that the “other” category is not considered an artifact category, as mixed signal or mixed signal and artifact components may constitute this category (see Pion-Tonachini et al., 2019).

In some (pre-)processing routines (mostly ERP routines), at this point or at the processing step 9, there is an additional segmentation in very small data segments and an additional artifact selection is performed after the IC cleaning procedure. We have mentioned it in this script during step 9 as a “revisited step 5” with code from the previous step 5 and not as an extra step, but if needed in your data, please inform the reader what segmentation steps have been taken and which statistical selection criteria has been used. Please avoid selection “by hand,” as in the previous step 5.

#### Step 8: Transform via Re-reference or Current Source Density (CSD)

**FIGURE 9:**
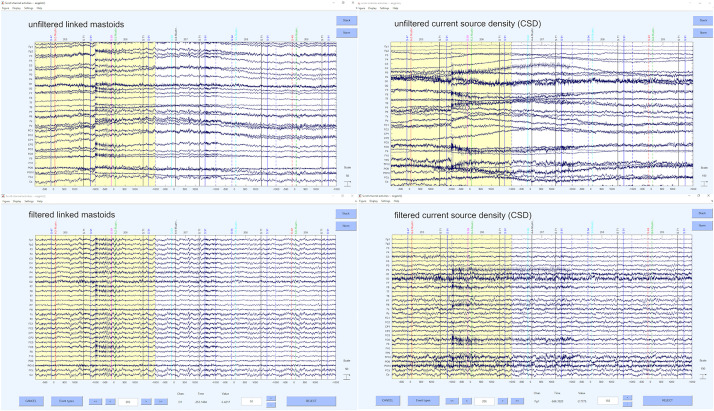
Unfiltered and filtered CSD transformed and mastoid referenced data solution.

Finally, we re-reference our data in the last step of the preprocessing. The selection of reference or montage is paramount to being able to visualize effects of interest, and the choice may be determined, in part, based on the standard of practice in your research domain and the specific question of interest. To the extent that spatially specific effects are important, the current source density (CSD) transformation is preferred and suggested by us (Hagemann, 2004; Rodrigues et al., 2021). Nevertheless, one may also choose another reference like linked mastoids or other reference schemes that are suitable and common in the respective field of EEG research. CSD provides an estimation of relative current at a point on the scalp surface as a function of the surrounding points. The distances are weighted with the relative activity on the electrode: The surface is estimated as a sphere, the signal differences to the adjacent are measured and the weighting of each difference is performed for each distance. Thus, a reference without reference is obtained and any electrode can be used. This results in a spatial filter that sharpens the topography of the (in)activation. There are two readily available possibilities for doing this, either the CSD toolbox from Kayser and Tenke (2006a, b) and Kayser (2009) or the *laplacian*_*perrinX* function provided by Cohen (2014), based on the approach of Perrin et al. (1990, 1989). These two tools provide comparable results, although the latter is substantially faster in execution. Nonetheless, we highly recommend visiting the website provided by Kayser^3^ in order to get more information about how CSD transformation is implemented and what it does. Alternatively, any other reference can be used with the command *pop_reref* (e.g., linked mastoids). As mentioned above, we perform this re-referencing at this rather late point, because of the automatic IC- detection. Examples of the unfiltered and filtered solutions for CSD and linked mastoid reference can be seen in Figure 9.

### Processing According to EPOS

#### Step 9: Segment the Data for Analysis

The goal of this step is to create a 4-dimensional matrix for the signal and each frequency for data analysis and to generate a 5-dimensional matrix for single trial analysis (of course, other dimensions in addition to the following dimensions can be generated). Depending on the size of the matrix, however, generating this matrix can lead to memory problems. A solution for this is to create only one matrix at a time or to resample the data during pre-processing as a very first step. To avoid estimated values based on interpolation, we advise resampling only to a new sampling rate that is a divisor of the previous one. Also, it is necessary to use anti-aliasing filters prior to resampling to avoid the introduction of aliased frequencies. To perform the resampling, there is for example the functions *pop_resample.* Generally, we would recommend recording the data only with the required sampling rate for all the planned frequency analysis and filtering (normally 250 Hz is sufficient) instead of the highest available recording frequency, to avoid resampling. Higher sampling rates, in unique cases, may help to overcome very specific data corruption problems, but normally they just take recording resources as well as lead to down-sampling of the data later, which could be avoided if sampled in a lower frequency right away.

At this point the data can be segmented again according to the relevant markers for the respective task to allow smaller segments to be extracted from the existing epochs if desired. As the first “segmentation” in the preprocessing was made with the intend to capture segments that are very fitting for IC decomposition and artifact detection and therefore might be overly long for a frequency response of interest or an event related potential, a second segmentation can be performed, now with the goal of getting a fitting epoch for data analysis. These markers must be selected, for which a separate segmentation script is recommended. An example of such a segmentation script is also provided along with the (pre-)processing chain. In this script, the variable “casearray” contains all relevant condition triggers for this experiment, grouped by condition. As mentioned above, in some processing pipelines (mostly ERP related) there is a second bad segment detection step at this point (see Step 5 pre-processing). Feel free to execute this step if needed and mention the necessary details. As a next step, the baseline correction is calculated automatically, however not with the *pop_rmbase* function from EEGLAB as we have encountered problems with the *round* function in different MATLAB versions, leading to wrong baseline applications in some MATLAB versions. Therefore, we avoid such compatibility issues by automatic calculation via script. Note, that the baseline taken here is used for either single trial analysis or mean analysis. Next, the frequencies of interest are defined. We assume that only specific time-frequency windows and ERP components will be analyzed in a hypothesis-driven fashion for the research question (of course, other frequency bands and ERP components can be considered for exploratory purposes). If desired, a filter with specific characteristics can be applied to the data depending on the ERP of interest. Finally, the user decides whether to look at single-trial data. We recommend using multilevel models for such a single-trial analysis. We also recommend using frequency bands instead of pure ERP-related data, since they have a higher reliability in single trial EEG analyses compared to similar single trial ERPs (Rodrigues et al., 2020a).

#### Step 10: Drop Conditions That Are Not Present

This step is short and simple. We recommend excluding those conditions (to reduce the amount of data) that are not contained in the data set but were present in the segmentation file. This is especially relevant for free choice paradigms, as some participants may have chosen not to act in a specific manner. Therefore, these cases can be dropped from the segmentation file for this person.

#### Step 11: Automatic Peak Detection in a Time-Window in EEG Signal

In this step, a peak is searched for in a time window of interest at an electrode position of interest via the averaged signal or the average over distinct conditions, leading to averages over trials in the respective condition instead of a total average. Please note, that the peak is not taken as a single value, but a time window is defined around the peak in order to avoid biases due to peak latency or artifacts and therefore capitalization on noise (Luck, 2005). In later steps we visualize and export the average of the trials in a condition or even the single trial values of the data in case of the intend to perform single-trial analysis. In case of single trial analysis, however, the single trial matrices need to be preprocessed first (Rodrigues et al., 2020a). The corresponding parameters (search window, electrode) depend on the ERP of interest. As a comment of literature recommendations, they are very important, as the ERP of frequency response of interest has normally investigated before and specific paradigms may provide specific physiological responses. However, the recommendations, guidelines and research propositions should be critically evaluated (e.g., it should be questioned whether the FRN is considered only at Fz, as is often the case in the literature due to earlier electrode montages, although FCz is also available and the respective topography also indicates that the component is rather mid-fronto-central than only limited to frontal regions). Hence a “standard” might be questioned by an informed decision in this step, partly aided by confirmation of the signal distribution.

#### Step 12: Compute and Visualize Event-Related Potentials

**FIGURE 10:**
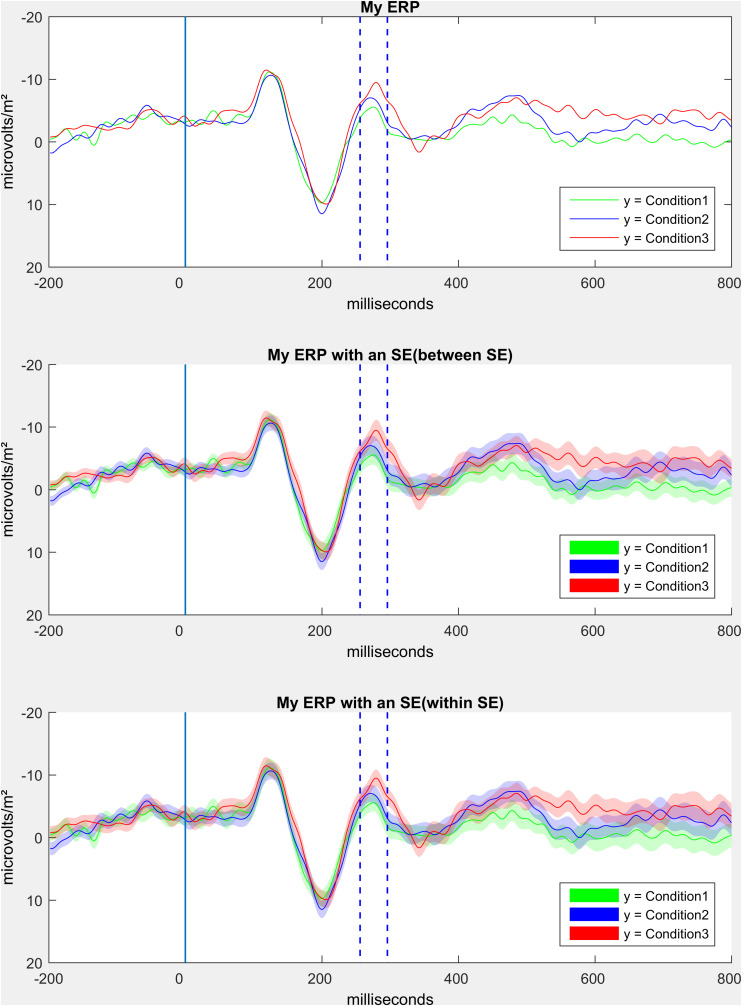
Comparison of the three different ERP plot options.

In this part of the script we offer the possibility to generate different forms of ERP graphs. First of all, an ERP can be plotted as it is traditionally seen in older manuscripts, only consisting of one waveform per condition (see Figure 10 upper panel). Next, we offer ERPs with shaded error lines (Kearney, 2020), which in addition to the course of the ERP also provide information about the precision of the estimate of the mean value (ERP). In this step, between errorlines (between standard error) are added to the figure (see Figure 10 middle panel). Alternatively, we provide code to add the mean within error lines (mean within standard error, being mean within standard errors of the differences of relevant conditions) to the figure (see Figure 10 middle panel). For the latter, it is important that the researcher is aware that meaningful conditions should be taken, or meaningful clusters of conditions should be calculated (only a short example in the script, but simply use the “nanmean” command).

#### Step 13: Topographical Maps (Topoplots) in the Time-Domain

**FIGURE 11:**
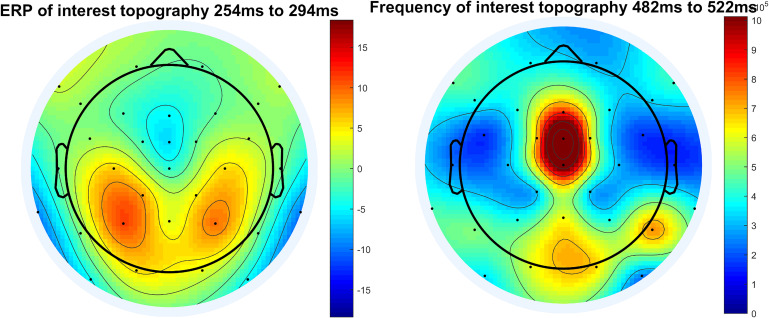
Topographical plot of ERP and topographical plot of frequency of interest, here example theta frequency and display of raw power.

In the next step we provide code to create topographic maps. We include the option to generate a topoplot for a time window of interest (peak-window) for ERP (see Figure 11), but also to create an animated graphics interchange format (GIF). This GIF depicts different time intervals to show the dynamic changes in the topography and to verify the selected time-window of interest as correct for the corresponding electrodes.

#### Step 14: Automatic Peak Detection in a Time Window in Frequency Band

We use morlet wavelets to perform time frequency decomposition. Our processing approach assumes the user has an *a priori* frequency band of interest and that the analysis focusses on said frequency band to avoid capitalization on chance findings.

Like the peak-detection in ERPs, the peak is searched for in the time-window of interest at the electrode of interest for the frequency band of interest. The corresponding parameters can be set in the same way as those used for the ERPs. The respective parameters should be taken from literature with critical view as mentioned above. Depending on the task, it might be useful to look at the average of all conditions (Cohen, 2014) or at the peak in certain conditions that differ from others, which, however, biases the chance to find significance. The attached script contains examples, but they must be adapted for different experiments.

#### Step 15: Topographical Maps for Frequency Responses

This step is identical to step 5, both the topographic maps for the peak-window of the frequency response and a GIF for the time course are implemented in the script. This is done to validate the choice of electrode of interest and the time window of interest (see Figure 11).

#### Step 16: Time-Frequency Plot for a Specific Electrode in a Broad Frequency Window

**FIGURE 12:**
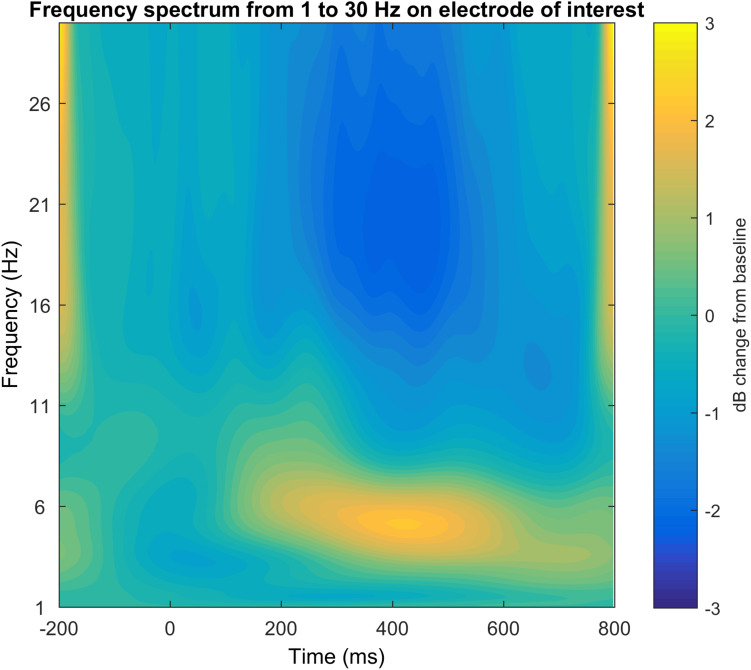
Time-frequency plot on the electrode of interest in the frequency spectrum 1-30 Hz.

In the last step of the graphical illustration of electrophysiological data, we implemented code for the creation of time-frequency plots. For this time-frequency plot we use the plot function based on the code provided by Cohen (2014) and edited by John J.B. Allen and Johannes Rodrigues (see Figure 12). It provides either a log transformed power output, a raw data output or the recommended dB change to baseline output that corrects for the power law that affects the display of different frequency bands together. The result is this time-frequency plot shows the frequency response not limited to the desired functional frequency but in a larger frequency windows in order to not only validate the selection of the electrode of interest and time window, but also the frequency of interest. As a recommendation, we suggest the spectral range of 1–30 Hz if you are not particularly interested in gamma frequencies. We also recommend using the dB to baseline change setting as mentioned above.

Concerning the gamma frequency band, we are rather cautious in interpretation and try to avoid it as there is evidence that microsaccades (Dimigen et al., 2009; Hipp and Siegel, 2013) and electrical muscular activity (Whitham et al., 2007) may drive these frequency spectrum responses.

#### Step 17: Export the Data to Statistical Software

In the last step of the processing “chain,” we offer code for exporting the EEG data into different statistical programs using the Excel (.xlsx) or the MATLAB (.mat) data formats. We support the export into the long format (i.e., each row represents a data-point in a specific condition combination per participant, with columns indicating the data as well as the conditions and the participants, resulting in multiple rows per participant) for the mean signals/frequencies. This format is for example required by many R (R Core Team, 2020) packages to calculate analysis of variance or multilevel analysis. SPSS (IBM, Armonk, NY) also requires long formatted data if a multilevel analysis should be performed. Furthermore, we offer the export into the wide format (i.e., all responses of a participant are in one row and each column represents the condition combination of the relevant data variable) for the mean signals/frequencies, which is used for example by Jamovi (The jamovi project, 2020) or in SPSS (IBM, Armonk, NY) to calculate analysis of variance. Finally, we also offer the export of single-trial signal/frequency data in long format, which is required by R or SPSS to calculate multilevel mixed models.

### Note Concerning Single-Trial Analysis

In this (pre-) processing chain, the opportunity of processing single trial data is provided although it is not the default option, but rather commented out to be used if activated. This was done to have some decent management over the necessary resources when using the processing pipeline the first time. Nevertheless, we want to encourage further exploration of the data and encourage the analysis of single-trial EEG responses (see e.g., Rodrigues et al., 2018, 2021) because of the interesting time dynamics that may happen in data that are mostly hidden if only mean responses over all trials are considered. These trial-level responses may provide information about learning, boredom as well as surprise and fatigue. They are very helpful in understanding the data and its implications in a better and maybe more precise way than just looking at the means. Also, inter-individual differences may hide in the variance of the responses, showing persons that are rather prone to be bored or similar reactions concerning the mentioned variables. Exploratory data analysis has an important role, but it is of course important to let it be guided by hypotheses and preregistration and open admission what findings are hypothesized and which are exploratory.

### Note Concerning Missing Interesting Analyses in This Chain

In this (pre-) processing chain, only a few analyses are provided and many interesting analyses like evoked and induced frequency responses (Galambos, 1992; David et al., 2006), cross-frequency coupling (e.g., with phase-amplitude coupling, Canolty and Knight, 2010), frequency phase distributions (e.g., Busch et al., 2009) and deeper source analysis with LORETA (Pascual-Marqui et al., 1994; Pascual-Marqui, 1999) or similar algorithms are not included. Also, PCA based ERP peak detection (e.g., Kayser and Tenke, 2003; Dien, 2010) is not included. One reason to not include them was our goal to establish a very basic pre-processing chain, i.e., a standardized beginning on which anyone may build on. This chain can hopefully also be used by novices who try to get in touch with EEG and get inspired by the analyses to understand and get to know their data based on hypotheses, but also based on exploratory validation of established criteria. Of course, we also like to encourage to explore data in other and newer ways, but a standardized basic result for hypothesis testing should be the first step, after which the data exploration follows. Another reason for not including some of the techniques mentioned above was the concern of introducing methods that are not that easy to understand and that may just be used as a “black box” without trying to think about them and to validate the results. After having seen many “odd” topographies, frequency response patterns and ERPs with questionable time-windows, we wanted to provide a standardized script that everyone is able to understand and is able to validate their results quickly. Nevertheless, all suggestions that have been made in these scripts should also be seen with caution, as they may not be the appropriate decisions for every data. The intention of this standardized approach is to provide researchers with a decent starting point of analysis, that can be modified and adjusted to their needs, to get to replicable and transparent analyses, if the respective changes are reported additionally.

### Measuring the Performance of the EPOS Pipeline

In order to measure the performance of EPOS pipeline on a sample dataset, we followed the idea of computing several metrics that were used to evaluate the HAPPE (Gabard-Durnam et al., 2018) on a long trial dataset of a virtual T-maze (see Rodrigues, 2016; Rodrigues et al., 2018, 2020b,c, 2021). Similar to their approach, we provide our sample dataset (Rodrigues and Hewig, 2021), so that users may evaluate the pipeline and possibly compare it to their own pipeline. In addition, we also provide a comparison to other processing pipelines using the Infant Sibling Project: Sample Files (Levin et al., 2017).

The metrics used to evaluate the processing pipelines were:

1) Channels that are not rejected (contributing “good” channels)
2) Rejected ICs (EPOS: After second ICA)
3) Variance kept after the rejection of the ICs (EPOS: After second ICA)
4) Number of rejected segment (EPOS: “Step 5” and “Step 5 revisited” combined)
5) Artifact probability of retained components (EPOS: After second ICA from IClabel)

The results for our own sample dataset can be seen in Table 2. While the channel rejection and the number of rejected epochs are rather inclusive, the variance of the dataset is rather restricted (see Table 2). However, the probability of retaining artifact components is rather small. Hence, we conclude that the EPOS is an inclusive approach concerning the general data rejection, with still a relatively low probability concerning artifacts. As mentioned in the scripts, EPOS was developed with having good recording quality as a necessary component for EEG research in mind. However, it may not only perform reasonably in exceptionally well recorded data, as can be seen below in the comparison to other pipelines for the Infant Sibling Project: Sample Files (Levin et al., 2017).

**TABLE 2:** Parameters of the performance on the sample dataset.

**Filename**	**Percentage channels kept**	**Number of epochs rejected in step 5**	**Number of epochs rejected in step 5 revisited**	**Percent independent components rejected**	**Percent variance kept after rejection**	**Median artifact probability in retained components**	**Mean artifact probability in retained components**
VP_02	89.23	0	0	0.53	17.70	0.005	0.017
VP_03	89.23	2	0	0.52	25.68	0.001	0.016
VP_04	86.15	0	0	0.46	20.66	0.003	0.011
VP_05	86.15	2	0	0.50	31.19	0.001	0.015
VP_06	95.38	1	3	0.55	28.74	0.001	0.015
VP_07	90.77	2	1	0.37	59.76	0.001	0.018
VP_08	89.23	3	0	0.40	28.67	0.002	0.016
VP_09	80.00	3	0	0.56	26.09	0.002	0.012
VP_10	83.08	0	0	0.54	21.72	0.001	0.013
VP_11	89.23	0	0	0.62	32.29	0.002	0.015
VP_12	87.69	2	0	0.54	31.42	0.003	0.021
VP_13	87.69	0	0	0.56	22.08	0.004	0.018
VP_14	87.69	0	0	0.46	15.90	0.001	0.009
VP_15	87.69	2	0	0.58	7.22	0.003	0.022
VP_16	87.69	0	0	0.49	20.47	0.003	0.023
VP_17	87.69	2	0	0.74	14.95	0.004	0.027
VP_18	87.69	0	0	0.72	19.99	0.005	0.023
VP_19	87.69	2	0	0.75	9.11	0.004	0.015
VP_20	87.69	4	0	0.51	11.46	0.002	0.016
VP_21	83.08	1	0	0.52	31.24	0.001	0.012
VP_22	92.31	1	0	0.62	18.76	0.002	0.013
VP_23	86.15	0	1	0.77	8.08	0.004	0.023
VP_25	87.69	1	0	0.63	24.14	0.002	0.027
VP_26	89.23	2	0	0.53	37.32	0.002	0.013
VP_27	84.62	1	0	0.60	17.73	0.002	0.018
VP_28	93.85	0	0	0.69	12.38	0.001	0.011
VP_29	89.23	1	0	0.71	13.41	0.003	0.020
VP_30	86.15	0	0	0.46	35.09	0.001	0.016
VP_31	87.69	3	0	0.58	7.64	0.002	0.017
VP_32	83.08	1	4	0.59	22.82	0.001	0.017
VP_33	89.23	0	0	0.55	16.01	0.002	0.015
VP_34	86.15	1	0	0.73	6.53	0.002	0.021
VP_35	87.69	1	0	0.56	29.29	0.005	0.025
VP_37	89.23	0	0	0.59	20.91	0.005	0.031
Dataset average	87.74	1.12	0.26	0.57	21.95	0.00	0.02
Dataset standard deviation	2.97	1.12	0.86	0.10	10.81	0.00	0.01

### Comparing EPOS and Existing Pipelines

To compare the EPOS with other pipeline approaches, we compared the metrics that were evaluated and published with HAPPE (Gabard-Durnam et al., 2018) to their given dataset (Levin et al., 2017) for a more objective comparison than a single performance on a more specialized dataset. We used the same 39 Channels of interest with average referenced data as the HAPPE (Gabard-Durnam et al., 2018) and chose to use the smallest suggested epoch length of 9 s (8 s with −1 s for baseline or filter data buffer) in the “first segmentation” (see step 2), including all data as we set a marker every 8 s in the data. Based on the results provided in the HAPPE manuscript (Gabard-Durnam et al., 2018), we were able to assess the Rejected ICs, the variance kept after the rejection of the ICs and the artifact probability of retained components.

The results are displayed in Table 3.

**TABLE 3:** Percentage of rejected independent components, percentage of variance kept, mean and median artifact probability of retained components of different preprocessing chains according to Gabard-Durnam et al. (2018) including the EPOS preprocessing chain on the Infant Sibling Project: Sample Files (Levin et al., 2017).

**Percent independent components rejected**	**HAPPE**	**ICA**	**Manual**	**ASR**	**ADJUST**	**SASICA**	**FASTER**	**FASTER-MARA**	**EPOS**
BaselineEEG01	50	97.37	92.11	82.86	26.32	44.74	5.26	94.44	85.29
BaselineEEG04	38.89	80.56	72.22	64.1	38.89	38.89	2.7	63.89	54.29
BaselineEEG05	37.14	45.71	37.14	53.85	8.57	20	5.26	38.89	35.29
BaselineEEG06	2.94	82.35	82.35	81.58	17.65	26.47	2.63	70.27	76.47
BaselineEEG07	2.78	97.22	100	86.49	11.11	11.11	5.41	85.71	72.22
BaselineEEG08	75.76	96.97	87.88	83.78	15.15	18.18	2.7	83.33	45.45
BaselineEEG09	71.43	97.14	74.29	79.49	62.86	22.86	2.7	86.11	48.48
BaselineEEG10	43.75	78.13	53.13	61.54	53.13	56.25	2.7	83.33	34.29
BaselineEEG11	35.48	90.32	48.39	74.36	16.13	19.35	2.7	75	62.86
BaselineEEG12	62.86	97.14	88.57	94.44	54.29	37.14	2.63	94.59	50.00
Dataset average	42.10	86.29	73.61	76.25	30.41	29.50	3.47	77.56	56.46
Dataset standard deviation	25.05	16.23	20.90	12.67	20.21	14.17	1.27	16.74	17.30
*p*-value of *t*-statistic to EPOS	0.145	0.001	0.053	0.006	0.005	0.001	0.000	0.009	1.

**Percent variance kept after rejection**	**HAPPE**	**ICA**	**Manual**	**ASR**	**ADJUST**	**SASICA**	**FASTER**	**FASTER-MARA**	**EPOS**

BaselineEEG01	48.28	1.9	4.21	50.59	85.9	63.13	96.1	4.17	8.44
BaselineEEG04	80.39	37.85	52.92	80.3	95.08	80.1	97.55	21.87	41.01
BaselineEEG05	79.08	74.28	77.48	69.27	78.61	87.49	93.89	80.12	50.10
BaselineEEG06	98.07	52.89	26.15	37.97	96.44	89.65	96.53	55.86	28.88
BaselineEEG07	99.13	2.5	0	18.88	95.42	91.87	89	24.91	29.86
BaselineEEG08	43.67	8.86	22.28	43.02	74.07	86.66	93.28	16.89	7.23
BaselineEEG09	24.25	7.56	30.38	33.58	78.77	77.17	93.28	16.26	6.28
BaselineEEG10	83.82	23.12	70.68	49.18	84.13	62.04	93.4	17.93	4.40
BaselineEEG11	85.15	37.73	59.9	42.41	79.79	89.89	95.75	41.54	2.31
BaselineEEG12	35.67	1.33	11.89	13.53	96.86	75.96	94.46	2.39	21.02
Dataset average	67.75	24.80	35.59	43.87	86.51	80.40	94.32	28.19	19.95
Dataset standard deviation	27.15	25.17	27.86	20.37	8.74	10.86	2.40	24.29	16.88
*p*-value of *t*-statistic to EPOS	0.000	0.620	0.150	0.011	0.000	0.000	0.000	0.391	1

**Mean artifact probability of retained components**	**HAPPE**	**ICA**	**Manual**	**ASR**	**ADJUST**	**SASICA**	**FASTER**	**FASTER-MARA**	**EPOS**

BaselineEEG01	0.14	0.25	0.41	0.18	0.41	0.28	0.87	0.35	0.04
BaselineEEG04	0.16	0.2	0.15	0.2	0.27	0.3	0.63	0.22	0.03
BaselineEEG05	0.08	0.28	0.21	0.25	0.35	0.35	0.46	0.25	0.02
BaselineEEG06	0.05	0.21	0.36	0.3	0.03	0.03	0.67	0.31	0.05
BaselineEEG07	0.05	0.45		0.26	0.04	0.04	0.79	0.36	0.03
BaselineEEG08	0.21	0.13	0.23	0.32	0.66	0.69	0.82	0.3	0.03
BaselineEEG09	0.15	0.26	0.21	0.25	0.52	0.63	0.82	0.3	0.02
BaselineEEG10	0.16	0.1	0.24	0.14	0.13	0.14	0.82	0.31	0.02
BaselineEEG11	0.07	0.35	0.25	0.33	0.23	0.23	0.71	0.23	0.04
BaselineEEG12	0.23	0.12	0.33	0.15	0.47	0.57	0.89	0.27	0.02
Dataset average	0.13	0.24	0.27	0.24	0.31	0.33	0.75	0.29	0.03
Dataset standard deviation	0.06	0.11	0.08	0.07	0.21	0.24	0.13	0.05	0.01
*p*-value of *t*-statistic to EPOS	0.11	0.00	0.00	0.00	0.01	0.01	0.00	0.00	0.00

**Median artifact probability of retained components**	**HAPPE**	**ICA**	**Manual**	**ASR**	**ADJUST**	**SASICA**	**FASTER**	**FASTER-MARA**	**EPOS**

BaselineEEG01	0.1	0.25	0.43	0.19	0.26	0.11	0.93	0.35	0.02
BaselineEEG04	0.16	0.2	0.12	0.18	0.2	0.19	0.78	0.29	0.01
BaselineEEG05	0.01	0.34	0.18	0.26	0.1	0.1	0.41	0.28	0.00
BaselineEEG06	0.03	0.22	0.43	0.27	0.03	0.02	0.76	0.38	0.01
BaselineEEG07	0.05	0.45		0.24	0.04	0.05	0.89	0.34	0.02
BaselineEEG08	0.25	0.13	0.2	0.28	0.76	0.77	0.94	0.32	0.01
BaselineEEG09	0.08	0.26	0.2	0.23	0.46	0.83	0.94	0.36	0.01
BaselineEEG10	0.12	0.1	0.31	0.03	0.08	0.08	0.95	0.36	0.01
BaselineEEG11	0.03	0.41	0.23	0.45	0.04	0.03	0.86	0.22	0.02
BaselineEEG12	0.18	0.12	0.33	0.15	0.37	0.56	0.96	0.27	0.01
Dataset average	0.10	0.25	0.27	0.23	0.23	0.27	0.84	0.32	0.01
Dataset standard deviation	0.08	0.12	0.11	0.11	0.24	0.32	0.17	0.05	0.01
*p*-value of *t*-statistic to EPOS	0.01	0.00	0.00	0.00	0.02	0.03	0.00	0.00	0.09

*The artifact probability for the EPOS chain has been computed using the IClabel classifications with the mean and median of the probability of all artifact components.*

In the context of the Infant Sibling Project (Sample files of Levin et al., 2017), the EPOS processing is a rather strict processing chain concerning the limitation of the variance, as the remaining variance is very limited compared to other approaches (see Table 3). However, the percentage of rejected components is neither rather high nor low and the probability of the artifact components is lower in almost every case (see Table 3), although it must be admitted that in the case of EPOS the calculation was based on the IClabel instead of MARA used in the other pipelines. Thus, we conclude that the EPOS may perform in a similar but more variance restricting and even less residual artifact prone fashion than the other example pipelines in this specific dataset (Levin et al., 2017).

## Discussion

We presented a standardized, automated open-source processing pipeline for EEG data. In times where replicability and standardization are becoming more and more important to increase the robustness of research results, we have presented a suggestion of a pipeline for (pre-)processing of EEG data as well as for detecting and graphically illustrating measured values, as a way to check the integrity of the processing results. We hope that the scripts included here will provide a basis to easily understand and replicate EEG analysis of future studies, as well as encourage people to explore their data and validate their results. In addition, an open and replicable pipeline may ensure that data sets from different sources could be transferred more easily into a joint analysis. The presented pipeline is not limited to ERP or frequency analysis but offers necessary code for both analyses and even single trial analysis. Nevertheless, it should still be mentioned, that this pipeline is merely a suggestion and may be adjusted to the respective needs of the data and paradigm. With this tool to get started with EEG data processing, users might hopefully develop a standardized and inspired way into analyzing the data and present valuable results to the scientific community.

Future developments and additional validations of this pipeline should compare clinical with healthy subjects and also cover different age ranges. In general, it might be worthwhile for future work to contrast the growing number of preprocessing pipelines in a review paper and evaluate the applicability and quality for specific subgroups.

## Data Availability Statement

The data used for the quantification of the performance of the EPOS is publicly available. This data can be found here: https://zenodo.org/record/4697026.

## Ethics Statement

The studies involving human participants were reviewed and approved by the Julius-Maximilians University of Würzburg. The patients/participants provided their written informed consent to participate in this study.

## Author Contributions

JR: conceptualization, data curation, formal analysis, funding acquisition, investigation, methodology, project administration, validation, visualization, writing–original draft: scripts and manuscript, and writing–review and editing: scripts and manuscript. MW: writing–original draft: manuscript and writing–review and editing: manuscript. JH: resources, software, supervision, and writing–review and editing: manuscript. JA: conceptualization; methodology, supervision, and writing–review and editing: manuscript. All authors contributed to the article and approved the submitted version.

## Conflict of Interest

The authors declare that the research was conducted in the absence of any commercial or financial relationships that could be construed as a potential conflict of interest.

## Footnotes

1 https://sccn.ucsd.edu/wiki/Chapter_01:_Rejecting_Artifacts

2 https://osf.io/cw5qv/

3 http://psychophysiology.cpmc.columbia.edu/software/CSDtoolbox/tutorial.html
